# Gender roles and constraints in enhancing hybrid chicken production for food security in lower Eastern Kenya

**DOI:** 10.1371/journal.pone.0318594

**Published:** 2025-03-03

**Authors:** John K. Musyoka, Wilckyster N. Nyarindo, Robyn Alders, Hezron N. Isaboke

**Affiliations:** 1 Department of Agricultural Economics and Extension, University of Embu, Embu Kenya; 2 Development Policy Centre, Australian National University, Canberra, Australia; National Museums of Kenya, KENYA

## Abstract

The adoption of hybrid chicken production as a source of income and food security among smallholder farmers has recently taken significant global attention. However, the effect of adoption of hybrid chicken across different genders on household food expenditure and food security remains unknown. This study employed propensity score matching (PSM) and endogenous switching regression (ESR) models to analyze the effect of adoption of hybrid chicken on smallholder households’ consumption expenditure and food security in Machakos County. The PSM and ESR models were estimated on a sample of 582 households selected using multistage stratified random sampling method. The descriptive results indicated that adopters of hybrid chicken spent on average KES 1296 compared to the non-adopters who spent KES 1400 on food consumption per month. Furthermore, the adopters of hybrid chicken were more food secure compared to the non-adopters. About 74% of female were also likely to experience chronic food insecurity compared to their male counterparts. The econometric results indicated that farm location, access to credit, access to market, access to feeds, group membership and cost of feeds significantly influenced the adoption of the hybrid chicken production for both male and female decision makers. The average treatment effect results showed that non-adopters spent higher costs on food consumption. Moreover, half of the adopters of hybrid chicken were food secure compared to the non-adopters. The treatment effects of adopting hybrid chicken on household food consumption and food security were positive suggesting that adopters benefited more compared to the non-adopters. To boost food security, this study recommends the National and County governments to develop a facilitating policy environment which supports chicken production systems that are socially, environmentally and economically sustainable while enhancing appropriate technical and extension support to enable cost effective chicken production, marketing, and disease control.

## 1. Introduction

In most developing countries, chicken is one of the predominant livestock species raised by both men and women in rural areas [1–4]. In Kenya, about 70 percent of the rural population rely on small-scale chicken farming for household food security and income generation [5,6], keeping over 31 million birds out of which 25 percent consist of hybrid chicken such as Sasso, Rainbow Roaster, Kuroiler, Karlo Kienyeji, Isa Brown and Kenbro [7]. However, women face gender disparities in terms of accessing productive resources, credit, extension and land limiting their productivity and affecting food security status [7–10]. The case is not different in chicken value chains where women are the dominant players in chicken production and also perceived to be participating in a “women’s” enterprise [4]. The situation of gender inequality and a disregard for gender in chicken production have resulted to increasing levels of poverty and lower productivity in most developing countries [11–15].

Hybrid chicken farming is an important enterprise in wealth creation and bioavailable micronutrient supply in peri-urban settings of most countries across the world [16]. Hybrid chickens is defined as a genetic modification, resulting from crossing of two or more breeds for commercial purpose [17,18]. They are bred to achieve high-performance when all input requirements are met and, are produced by cross selected breeds to obtain specific desired traits [16,19]. In addition, these chickens are preferred birds by many smallholder farmers in peri urban areas for generating higher profitability due to their fast growth and higher body weight compared to indigenous chicken [20]. Furthermore, hybrid chicken remains the ideal enterprise to reduce protein deficiency experienced by humans in many countries [17]. Although extensive literature exists on different ways of improving hybrid chicken production among smallholder farmers [21,22], in Kenya a declining trend in hybrid chicken numbers has been recently observed due to high cost of feeds, high disease incidences, inadequate nutrition, and poor marketing channels [3,5,23].

Hybrid chickens have been reported to contribute significantly to the household economies by providing a source of regular income that can be used by households to buy necessities [24]. Additionally, women who raise small flocks of chickens can generate income fully under their control, contributing to their empowerment and the food security of their households. The conversion of various sources of potential human food to animal feed and particularly monogastric animals like chickens is a significant food security concern [25]. However, the scavenging feed resource base used in extensive chicken farming transforms environmental feed components into delicious and nutrient-rich food products for people [26]. Recent studies have focused mainly on basic improvement of chicken genetics to high yielding and early maturity [3,27], while little attention has been given to the contribution of hybrid chickens to household food consumption and food security [28–30].

The majority of smallholder farmers depend on chicken production, especially in low-and middle-income countries (LMIC), as part of their livelihood strategy [31]. The World Food Programme (WFP) indicates that the majority of LMIC still face chronic or acute food insecurity, which has a disastrous impact on their economies [32]. Food availability and accessibility are greatly impacted during prolonged crises and frequently manifests in households, altering livelihoods, nutritional status, and food systems [33]. Adoption of chicken farming, particularly hybrid chicken production in peri-urban areas has been seen as a key strategy for reversing matters focusing on rural and agricultural development [34]. In addition, hybrid chicken production has been identified as a means of improving livelihoods through provision of income from the sales of the surplus, thus creating employment as well as food and nutrition security [25,35,36]. Despite the foregoing benefits, this enterprise continues to face low and declining outputs contributing to increased food insecurity among the rural households in Kenya and a number of other countries, particularly due to increase in feed prices associated with increasing frequency of extreme weather events and political instability [36–39].

Household Food Insecurity (HFI) is defined as a situation that exists when members of household have inadequate diet either partially or throughout the year or face the possibility of inadequate diet in the future [40]. HFI is a common problem constraining rural livelihoods in regions where most small-scale chicken production systems are practiced [41]. Food availability is one of the main constituents of food security, meaning food should be available at the national and household levels [42], and seen favorably by the community concerned both socially and culturally [7]. The majority of livestock in areas with limited resources are typically chickens, which contribute to the availability of food in two ways: directly by providing nutrient-rich and culturally acceptable products for human consumption, and indirectly by provision of manure and income to agricultural production [43].

Recent studies on determinants of adoption of different types of chickens have revealed that land ownership, age, distance to sealed roads, and off-farm income to be key factors influencing adoption of hybrid chicken among women [44,45]. Other studies have revealed that age, gender, inadequate knowledge, membership of chicken farmer groups, and number of extensions contacts also have a significant effect on chicken production by both male and female producers [30,46]. However, most of these studies failed to incorporate gender factors as well as the cost of feeds which play a key role in decision-making. Understanding how gendered roles affect household food security and women’s wellbeing is essential in pursuing sustainable development [47,48].

Women’s participation in agriculture has been widely documented, but there remains a need for more gendered data on women’s and men’s roles in different value chains, including hybrid chicken production. Evidence shows that women provide over 40% of agricultural labour and on top they typically have more domestic duties compared to men, limiting them to livestock value chains like marketing [49,50]. Thus, there is a substantial gender gap in the allocation of the proceeds of productivity between women and men managed enterprise [51]. The situation is exacerbated by current social norms and gender inequalities, with women facing more challenges in regard to access and control over productive enterprises such as hybrid chicken production [51].

A study on the contribution of chicken to household food security (HFS) in LMICs found that farmers who adopted hybrid chicken in their homesteads improved their HFS [30]. Chicken farming is also reported to have a positive and significant effect on HFS and nutritional status in a study conducted across East African countries [3]. Furthermore, adoption of hybrid chicken production among smallholders in Southeast Ethiopia had a positive effect on household food security [1,52]. A study on the effect of adoption of agricultural technologies on food consumption expenditure using ESR model found that food consumption expenditure per household was significantly higher for the non-adopters compared to their counterparts [53].

Despite the considerable literature on the connection between gender and agricultural productivity, most literature uses the household head as the proxy [9,54], and most of these studies exclude female farmers in Male-Headed Households (MHHs) who make the decisions regarding the chicken operation. The gender of the household head does not always serve as a perfect predictor of female access or decision-making across farm activities [55,56]. Therefore, this study seeks to answer the question “whether gender affects adoption of hybrid chicken and its impacts on household food consumption and food security”. This study answers this question by analyzing factors determining the adoption of chicken types based on the gender of the chicken farm decision-maker. Secondly, the study evaluates the factors constraining the adoption of hybrid chickens and its impact among households disaggregated as chronically food insecure, transitorily food insecure, break-even food secure and food surplus among smallholder chicken farming households in Machakos County.

## 2. Materials and methods

### 2.1. Study area

**Fig 1:**
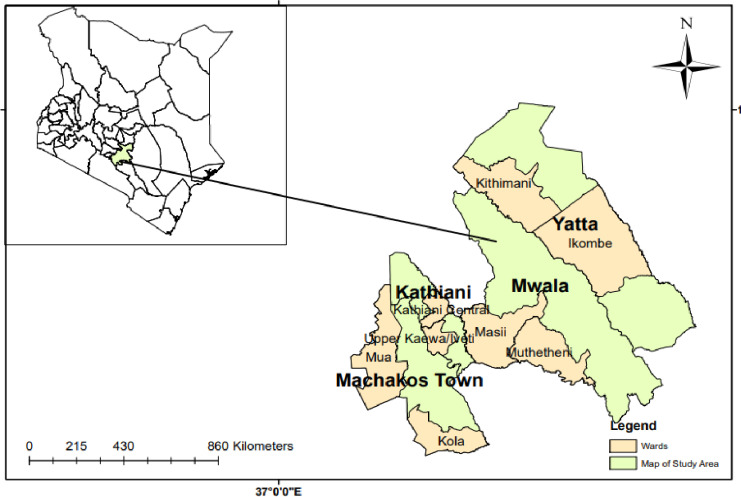
Map of Machakos County and the sampled wards.

The study was conducted in four sub-counties (Kathiani, Machakos town, Mwala and Yatta) of Machakos County (Fig 1). The County is located between latitudes 0^o^45’ and 1^o^31’ South and longitudes 36^0^45’ and 37^o^45’ East. A report by [57] show that the county has a total population of 1,421,932 people. In addition, the county lies in lower midland agro-ecological zone. The annual rainfall in the area ranges between 500 to 1300 mm with temperatures ranging from 18°C to 25.7°C [58]. Due to high temperatures and limited rainfall experienced in the area, crop production has been significantly affected while livestock production has become a top priority. Chicken farming, as one of the livestock production subsectors, has been identified as that with the most potential in the area [59].

### 2.2. Sampling design and technique

The study used survey data collected between 10^th^ and 31^st^ October in 2022 from a sample of 582 chicken farming households in Machakos County. The County was purposively selected since hybrid chicken production has been widely disseminated through numerous stakeholders such as the County government, Agricultural Sector Development Support Program (ASDP) and Kenya Climate Smart Agriculture Project (KCSAP). Four out of eight Sub-counties namely; Kathiani, Machakos town, Mwala and Yatta in Machakos County were purposively selected since they were the pilot sites for the KCSAP project with the households stratified into project hybrid chicken farming and indigenous chicken farming [60]. Within each Sub-County, two wards were randomly selected to form the sampling frame with the list provided by the agricultural livestock extension officers. A simple random sampling procedure was used to select 151 hybrid chicken and 431 indigenous chicken farming households using the probability proportionate to size approach (Table 1). Following a study by [61] a household formed part of the sampling frame if the respondent had a minimum of 20 chickens at the time the survey was conducted. The threshold for the number of birds per household was key to be able to collect information on chicken management practices. The quantitative questionnaire was administered to the households to collect demographic, socio-economic and institutional information on hybrid and indigenous chicken farming households and their associated food consumption expenditure and food security status (S1 Appendix ).

**Table 1:** Target population and sample size of the chicken farming households per ward sampled.

County	Sub-Counties	Wards	Number of households	Households Sampled
Machakos	Kathiani	Kathiani central	13,812	71
		Upper Kaewa	13,192	68
	Machakos	Mua ward	13,992	72
		Kola ward	10,433	53
	Mwala	Muthetheni ward	11,276	58
		Masii ward	16,965	86
	Yatta	Kithimani ward	17,499	90
		Ikombe ward	16,372	84
	Total		113,541	Sample size = 582

**Source:** Ministry of Agriculture Livestock and Fisheries, Machakos County, (2022).

### 2.3. Ethics approval and consent to participate

Ethical clearance was obtained from National Commission for Science, Technology and Innovation (NACOSTI) with the license number NACOSTI/P/22/20050. In addition, formal letters of cooperation were written to the four Sub County Livestock Extension officers through the office of the County Livestock Extension officer in Machakos County. Verbal and written consent was obtained from all the participants before data collection. Privacy, anonymity and confidentiality were guaranteed throughout the process of the study.

### 2.4. Analytical framework

#### 2.4.1. Household food security and consumption measures.

Household food security was defined as the situation in which all household members always have physical, social and economic access to safe and nutritious basic food that meets minimum dietary needs and food preference for an active and healthy life. This study adopted the Food Agriculture Organization (henceforth FAO, (2022) approach for measuring food security status among households using food insecurity experience scale [7]. This approach measures the severity of food insecurity at the household level. The food insecurity experience scale (FIES) consisted of eight yes or no questions that inquired about the behaviour and experiences associated with food insecurity among household members. Following a study by [62], a quantitative assessment of food security among chicken farming households in Machakos County in the last 12 months at the time of the survey was conducted.

The respondents were asked to make self-assessment of the situation of household food security in the last twelve months based on all the available sources of food. The situations were further classified into a scale of 1 = none, 2 = rarely (1–2 times), 3 = sometimes (3–10 times) and 4 = often (>10 times). The respondents were further grouped into four mutually exclusive options including chronic food insecure (food shortage throughout the year); transitory food insecure (occasional food shortage); break-even (no food shortage but no surplus), and food surplus throughout the year (food secure). The household were later re-grouped into two; food secure comprising of breakeven and food surplus equivalent to 1 and food insecure equivalent to 0 comprising of chronic and transitory food insecurity households.

Furthermore, the study followed FAO, (2022) to collect household food consumption expenditure information [2]. This study analysed food expenditure and defined the term “consumption” as the purchase of foods, regardless of the end-use of what was purchased. Therefore, “food consumption” was referred as the intake of a food, possibly net of unusable parts and food consumption expenditure was measured in monetary form as the average amount used in purchasing food in a month per household.

#### 2.4.2 Propensity score matching (PSM) model.

To analyze the effect of adoption of hybrid chicken on household food consumption expenditure and food security, PSM was used. Following a study by [63] a two stages analysis was adopted. In the first stage, a probit model was used to estimate the socio-economic and demographic factors that influenced the probability of adoption of hybrid chicken based on the gender of the main decision maker of the enterprise. Following [63], the model was expressed as;


$$Y_{1}=\beta_{1}X+\varepsilon_{1}Y_{0}=\beta_{0}X+\varepsilon_{0}$$ 1

where $Y_{1}$ is equivalent to adopters of hybrid chicken and $Y_{0}$is the non-adopters representing indigenous chicken farming households, *X* are the observable characteristic for both groups and $\varepsilon_{1}$ and $\varepsilon_{0}$ is the error term. `

In the second stage, PSM approach was used to evaluate the effect of adoption of hybrid chicken on food consumption expenditure and household food security among chicken farming households. The PSM addressed the potential selection bias by comparing adopters of hybrid chicken and non-adopters representing indigenous chicken farming households [64]. Following [63], the net impact of the PSM was expressed as;


$$netimpact=Y_{1}-Y_{0}$$ 2

where$Y_{1}$ represents the treated group (adopters of hybrid chicken) and $Y_{0}$ is the un-treated group (non-adopters representing indigenous chicken farming households).

#### 2.4.3 Nearest neighbor matching and average treatment effect.

The Average Treated Effect (ATE) was estimated using the Nearest Neighbor Matching (NNM) technique [65]. This is one of the simplest matching approaches in estimating average treatment effects. A member of the comparison group is chosen as a match for a treated individual based on the closest propensity score (or the instance with the most similar observed characteristics). Untreated (ATU) individuals were matched more than once using nearest neighbor matching with replacement approaches. By using the nearest neighbor method, it is guaranteed that the most comparable observation will be used to build the counterfactual. In this study, the variables used to predict food consumption expenditure and food security were also associated with the likelihood of adopting hybrid chicken. To eliminate bias, [66] advocated matching the propensity ratings between treated and control groups. Treated group represented adopters of hybrid chicken while control group represented non-adopters of hybrid chicken. As a result, the use of NNM served to control the confounding and calculate ATT.

#### 2.4.4 Endogenous switching regression (ESR) model.

To check for the robustness of the PSM model and correction of the selection biases of unobserved factors, ESR model was used to examine the influence of hybrid chicken adoption on household food consumption and food security, with households facing two regimes: (1) adopters of hybrid chicken or (0) non-adopters. Following [67], the ESR model was expressed as;


$$\text{Regime 1}:Y_{1j}=\;X_{j}\beta_{1}\;+\;U_{1j}ifA_{j}=1\left(adopters\right)$$ 3a

$$\text{Regime 2}:Y_{1j}\;=\;X_{j}\beta_{2}+\;U_{2j}\;ifA_{j}=0\left(non-adopters\right)$$ 3b

where $Y_{1}$ is the household food consumption and food security in regimes 1 and 2, $X_{1}$ represents a vector variable that influences the net impact variables.

If a correlation exists between the error term and the outcome equation (3a) and (3b) and the adoption equation (1), estimating (3a) and (3b) without accounting leads to a biased estimate [67,68]. Thus, for hybrid chicken adopters and non-adopters, the outcome equation (food consumption expenditure and food security) corrected for endogenous adoption is presented as;


$$\text{Regime }1:\;Y_{1j}=\beta_{1}X_{1}+\sigma_{1\varepsilon }h_{1j}+n_{1j},ifA_{j}=1$$ 4a

$$\text{Regime }2:Y_{2j}=\beta_{2}X_{2}+\sigma_{2\varepsilon }h_{2j}+n_{2j},ifA_{j}=0$$ 4b

Equations (4a) and (4b) demonstrate the Inverse Mill Ratio (IMR) generated using the probit model of the selection equation to adjust for the selection bias in the second stage estimation. The parameters to be estimated are β and σ, and the error term *n* independently and identically distributed with mean zero and constant variance. The actual and counterfactual impact outcomes are specified below using the two regimes of the outcome equations, (4a) and (4b).


$$E\left(Y_{1}\text{|}X,A_{j}=1\right)\;=\;X_{1j}\beta_{1}\;+\sigma_{1\varepsilon }h_{1j}(\text{Hc adopters})$$ 5a

$$E\left(Y_{2}\text{|}X,A_{j}=0\right)\;=\;X_{2j}\beta_{2}+\;\sigma_{2\varepsilon }h_{2j}\left(\text{Hc non- adopters}\right)$$ 5b

$$E\left(Y_{2}\text{|}X,A_{j}=1\right)\;=\;X_{1j}\beta_{2}\;+\;\sigma_{2\varepsilon }h_{1j}\;(\text{Hc adopters had they decided not to adopt})$$ 5c

$$E\left(Y_{1}\text{|}X,A_{j}=0\right)\;=\;X_{2j}\beta_{1}\;+\;\sigma_{1\varepsilon }h_{2j}\;\left(\text{Hc non-adopter had they decided to adopt}\right)$$ 5d

The equations (5a) and (5b) represent the actual expectations observed in the sample, whereas (5c) and (5d) indicate the counterfactual outcomes. The average effect of treatment (adoption of hybrid chicken) on the treated (ATT) was obtained by subtracting equations (5a) and (5c).

## 3. Results and discussion

### 3.1. Descriptive statistics

#### 3.1.1. Household and farm characteristics disaggregated by gender of the respondent.

The descriptive summary statistics of the sampled households disaggregated by the gender of the main decision makers in chicken enterprise are presented in Table 2. Generally, the results show that female respondents (79%) dominate in decision-making of chicken production activities compared to the male respondents (21%). The t-test results indicate a significant difference in age of household head between male decision-makers (60 years) and female decision-makers (54 years). The mean number of years spent in school by most of the respondents was 11 years; an indication that majority of the smallholder chicken farmers in the study area have attained basic secondary education. The average mean size of the households were 5 people. Regarding resource constraints and access to the inputs, the mean total land size owned by the respondents in the study area was 4 hectares while the total farm size under agricultural practice including chicken farming was 3 hectares. Further, results indicated that 33% of the farmers had access to credit and this facilitated purchasing inputs such as feeds.

**Table 2:** Descriptive characteristics of the households disaggregated by gender of the main decision maker.

Variables	Pooled (n = 582)	M-DM (n = 124)	F-DM (n = 458)	T-test
Age of household head (years)	55.46 (0.54)	59.82 (1.21)	54.28 (0.59)	0.000***
Education (number of years spent in school)	11.09 (0.11)	11.45 (0.15)	10.99 (0.13)	0.081*
Household size (number of people)	5.04 (0.08)	5.12 (0.19)	4.77 (0.09)	0.085*
Total land size (hectares)	3.66 (0.12)	3.48 (0.24)	3.70 (0.13)	0.427
Farm size (hectares)	2.61 (0.08)	2.67 (0.19)	2.59 (0.09)	0.695
Access to credit (1 = yes, 0 = no)	0.33 (0.02)	0.28 (0.04)	0.35 (0.02)	0.174
Walking distance to input market (minutes)	0.54 (0.26)	0.56 (0.24)	0.48 (0.28)	0.652
Indigenous chicken (1 = yes, 0 = no)	0.85 (0.07)	0.28 (0.08)	0.72 (0.06)	0.378
Hybrid chicken (1 = yes, 0 = no)	0.42 (0.03)	0.35 (0.04)	0.53 (0.03)	0.001***
Group membership (1 = yes, 0 = no)	0.46 (0.03)	0.43 (0.06)	0.55 (0.05)	0.021**
Number of groups HH belongs to	1.54 (0.34)	1.43 (0.26)	2.64 (0.42)	0.035**

**Note:**

***,

** and

*  denotes 1%, 5% and 10% levels of significance respectively, M-DM is the male decision maker, F-DM represents the female decision makers, standard errors are in parentheses.

The average walking distance to the nearest input market was approximately 32 minutes. This suggests that the respondents were closely located near the input markets and were able to access the necessary inputs required in chicken farming. The results on the chicken enterprise indicated that 85% of the respondent practiced indigenous chicken farming. The t-test results further indicated a significant difference in the adoption of hybrid chicken between female decision-makers (53%) and the male decision-makers (35%). Furthermore, there was a positive and significant difference in group membership between the female decision-makers (55%) and the male decision-makers (43%) within the chicken enterprise. In addition, male decision-makers belonged to an average of one group while female decision-makers belonged to an average of three chicken farming groups existing in the study area (Table 2).

#### 3.1.2. Chicken production disaggregated by adopters and non-adopters of the hybrid chicken.

Table 3 presents the comparative assessment of the key variables for the adopters and non-adopters of hybrid chicken. The results indicate that 34% of the chicken farmers accessed credit with significant difference observed between the adopters (42%) and non-adopters (31%) of the hybrid chicken. The plausible reason is that adopters of technology are able to access credit since they own more assets including off farm income hence more easily financed by financial institutions compared to non-adopters [69,70]. Furthermore, about 58% of the adopters of hybrid chicken belonged to the farmer group compared to 43% of the non-adopters of hybrid chicken. Farmer groups act as a platform through which farmers access information on new agricultural technologies, access inputs and market their outputs [10].

**Table 3:** Descriptive characteristics of the households disaggregated by the adopters and non-adopters of hybrid chicken.

Variables	Adopters (151)		Non-adopters (431)		Pooled Data (582)		
Categorical variables	Freq	Percent	Freq	Percent	Freq	Percent	χ2
Gender							
1 = Male	28	18.54	96	22.27	124	21.31	0.335
0 = Female	123	81.46	355	77.73	458	78.69	
Access to credit							
1 = Yes	63	42	131	30.75	194	33.68	0.012**
0 = No	87	58	295	69.25	382	66.32	
Location of the farm							
1 = Homestead	146	98.65	425	99.77	571	99.48	0.105
0 = Another site	2	1.35	1	0.23	3	0.52	
Group Membership							
1 = Yes	85	58.22	181	42.79	266	46.75	0.001***
0 = No	61	41.78	242	57.21	303	53.25	
Extension services							
1 = Yes	51	33.77	41	9.51	92	15.80	0.002***
0 = No	100	66.23	390	90.49	490	84.20	
Continuous Variables	Mean		Mean		Mean		t-test
Household head age	54.91 (14.23)		55.57 (13.08)		55.40 (13.38)		0.603
Education	10.45 (6.82)		8.90 (4.60)		9.24 (5.71)		0.001***
Household size	6.92 (8.33)		4.94 (1.93)		5.45 (9.50)		0.027**
Household income	5823. 1 (2360.2)		2639.9 (1870.4)		3465.8 (2842.5)		0.004***
Farm size (hectares)	2.61 (2.10)		2.62 (2.07)		2.62 (2.07)		0.954

**Note:**

***,

** and

*  denotes 1%, 5% and 10% levels significance respectively, standard errors are in parentheses, exchange rate at time of data collection 1USD = 120 Kenyan Shillings.

Additionally, about 16% of all respondents accessed extension services with adopters having a higher percentage of extension contacts (34%) compared to the non-adopters (10%). There was also a significant difference in the average number of years spent in schooling (χ2 = 0.001) between farmers who had adopted hybrid chicken (10 years) and those who did not (9 years). On average, the household size for the hybrid chicken adopters were significantly higher compared to the non-adopters of hybrid chicken. The possible reason is that household size is used as proxy for labour productivity in agricultural activities [10]. Furthermore, there were significant differences in the mean of the household income (χ2 = 0.004) between the adopters of hybrid chicken (KES 5,823.18) and the non-adopters (KES 2,640).

#### 3.1.3. Outcome and treatment variables.

The descriptive statistics showed that the average household food consumption expenditure for the total sampled households was KES 1323 with adopters spending less on food items compared to the non-adopters (Table 4). This implies that households with male and female managers who adopted hybrid chicken were better placed income-wise compared to non-adopters who could have diversified their range of food basket. Furthermore, about 49% of the male and female respondents were food secure with adopters of hybrid chicken being more food secure (50%) compared to the non-adopters of hybrid chicken (44%). In relation to food security categories, non-adopters of hybrid chicken where more chronically food insecure compared to the adopters. However, there were no notably significant differences between the adopters and non-adopters of hybrid chicken in transitory food insecurity, breakeven food security and food surplus (Table 4).

**Table 4:** Descriptive statistics of the outcome and treatment variables disaggregated by adopters and non-adopters of hybrid chicken.

Variable	Measurement	Pooled (n = 582)	Adopters n = 151	Non-adopters N = 431
Food Consumption	In Kenya shillings	1322.68 (3168.34)	1295.73 *** (155.57)	1399.62 (242.19)
Food Security	1 = Food secure	0.487 (0.020)	0.502 (0.024)	0.444 (0.041)
Chronic food insecurity	1 = Yes	0.040 (0.008)	0.033 (0.009)	0.060** (0.019)
Transitory food security	1 = Yes	0.473 (0.021)	0.465 (0.024)	0.500 (0.041)
Breakeven food security	1 = Yes	0.370 (0.020)	0.383 (0.023)	0.331 (0.038)
Food Surplus	1 = Yes	0.117 (0.013)	0.119 (0.016)	0.113 (0.026)

**Note:**

***,

** and

*  denotes 1%, 5% and 10% levels of significance respectively, standard errors are in parentheses, exchange rate at time of data collection 1USD = 120 Kenyan Shillings.

#### 3.1.4. Gender participation in hybrid chicken production activities.

**Fig 2:**
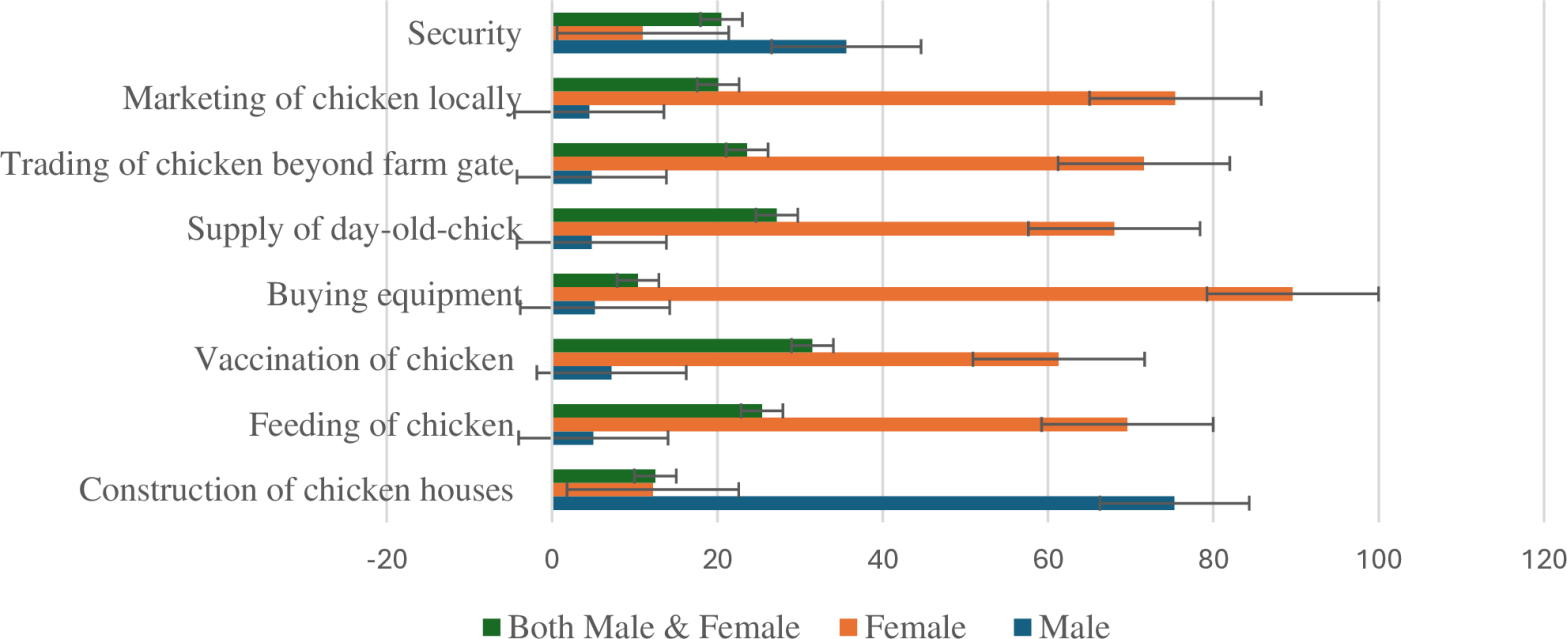
Chicken production activities disaggregated by the gender of the household.

Fig 2 presents the descriptive statistical results of gender roles in hybrid chicken production activities. Following studies by [71–73] gender roles was defined as the assessment of distinct activities, responsibilities, and decision-making authority assigned to men and women, shaping their interactions and influencing the ability to address constraints and optimize benefits in the chicken enterprise. The results indicate that majority of male farmers 75% participate in the construction of chicken houses. The results further showed a higher proportion of female farmers participated in hybrid chicken enterprises, the majority (76%) selling hybrid chickens to the local market.

About 72% of the female farmers traded the hybrid chicken beyond the farm gate with 68% of the female farmers participating in the supply of day-old-chick. The results also indicated that most female farmers (90%) were involved in buying of equipment, 70% engaged in feeding of chicken while 61% were involved in vaccination of hybrid chicken. This implies that chicken production is predominantly managed by women, hence, its recognition as a women’s enterprise.

From the results, it can be observed that female farmers dominate the marketing, trading, supply of day-old-chick, buying of equipment, vaccination as well as feeding of chicken. Furthermore, female farmers contributes to the bulk of agricultural sector in Kenya [8,74], though limited to productive resources including land, access to education, information, and financial resources [75,76]. However, gender participation in poultry value chain have indicated that male farmers dominates in decision-making regarding vaccinations, feed purchases, and selling of chicken since have control over household productive resources [51].

#### 3.1.5. Comparison between household head gender and chicken enterprise main decision maker to food security status.

**Fig 3:**
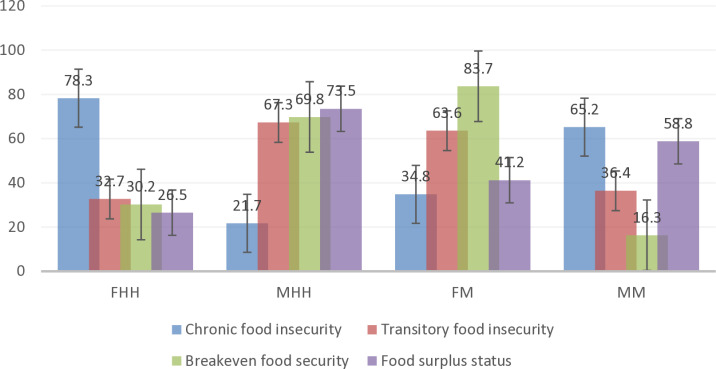
Food security status disaggregated by Female Household Head (FHH) verses Male Household Head (MHH) and Female hybrid chicken manager’s verses Male managers.

Fig 3 presents the findings showing the distribution of food security status for the Male Headed Households (MHH) versus the Female Headed Households (FHH) and the Female Managers (FM) versus Male Managers (MM) of the chicken enterprises. The results indicated that, holding all other factors constant, female headed household are likely to experience chronic food insecurity at 78%. This implies that females are still facing challenges of accessing productive resources such as credit, land ownership and social safety nets which are essential for securing adequate food supply. On the other hand, male-headed households are likely to achieve food surplus at about 74%. These results agree with the findings of [66] in Nigeria and Ethiopia, who found that female headed household are usually more food insecure compared to the male headed household.

In contrast, a study by [9] in Kenya, evaluated food security status among the male headed household and female headed household but found no significant difference in terms of food security between the two groups. With regards to the main decision maker in the chicken enterprise, about 84% of the female managed chicken enterprise were at breakeven food security while majority of the male managed chicken enterprise (65%) experienced chronic food insecurity.

### 3.2. Econometric results

#### 3.2.1. Evaluation of gendered determinants of adoption of hybrid chicken in Kenya.

Table 5 presents the results of the Probit regression model evaluating the determinants of adoption of hybrid chicken among smallholder farmers disaggregated by gender of the main decision maker in chicken enterprise in lower Eastern Kenya. The data was disaggregated to fill research gaps arising from previous studies of influence of gender on the adoption of agricultural technologies based on the main decision maker of the enterprise [51,77–79]. Overall, the results of the probit model were significant at 1% indicating that the data fitted the model well with high prediction power of chi square (LR chi2 (15) =  169.20, Prob> chi2 = 0.0000). Furthermore, Pseudo R^2^ of 30.68 indicated that 31% of the variation in the dependent variable was explained by the inclusion of the independent variables in the model. The results indicated that access to credit, location of the farm and cost of the feeds had a positive influence on the adoption of the hybrid chicken if the main decision maker in enterprise was male. On the other hand, group membership, access to credit, access to market and feeds had positive influence on adoption of the hybrid chickens if the main decision maker in the enterprise was a female, while feed cost o had a negative effect on the adoption of hybrids if the main decision maker of the enterprise was a female.

**Table 5:** Maximum likelihood estimates of probit model on the gendered determinants of adopting hybrid chicken in Machakos County.

	Male decision maker		Female decision maker	
Variables	ME(dy/dx)	RSE	ME (dy/dx)	RSE
Household size	0.242	0.015	0.114	0.027
Household head gender	‒0.032	0.181	0.075	0.136
Household head age	‒0.014	0.012	0.374	0.017
Number of years spent in school	0.235	0.037	0.138	0.002
Farm size (Hectares)	0.162	0.025	0.285	0.038
Household income	0.002	0.123	0.018	0.160
Use of the automated system	0.003	0.152	0.019	0.121
Location of the farm	0.042***	0.762	0.051	0.174
Access to the credit	0.060**	0.121	0.021**	0.142
Group membership	0.046	0.235	0.087***	0.138
Cost of extension contact	‒0.121	0.242	0.053	0.129
Access to feeds	0.201	0.231	0.151***	0.110
Cost of the feeds	0.015**	0.108	‒0.161***	0.245
Cost of vaccination	0.004	0.186	‒0.027	0.017
Access to market	0.009	0.136	0.127**	0.128
Distance to input market	‒0.108	0.072	‒0.030	0.214

**Note**:

***,

** and

*  denotes 1%, 5% and 10% levels of significance respectively, ME (dy/dx) is the marginal effects, RSE is the robust standard errors, number of observations = 582, LR chi2 (15) = 169.20, Prob> chi2 = 0.0000, Pseudo R^2^ = 30.68.

The location of the farm increased the probability of adopting hybrid chicken production if the main decision maker of the enterprise was a male by 4.2%. The possible explanation is that male are the main decision-makers within the household, especially on aspects of productive resources that deals with agricultural activities [80] and this may influence the adoption of new technologies or practices, with males exerting more influence over decisions related to production and investment areas due to social norms.

Access to credit had a positive association for the adoption of hybrid chicken production when the enterprise was either managed by male or female managers. This positive impact was higher for male managed enterprises compared to their female counterparts. One-unit increase in access to credit increased the probability of adoption of hybrid chicken by 6% and 2.1% for the male and female managed enterprises respectively. The plausible reason could be due to the fact that smallholder farmers can easily access credit thus better positioning them to invest in hybrid chicken breeds that are characterized by fast maturity and high yield. A cross-country study conducted in Ghana, Tanzania, and Kenya revealed a positive relationship between access to credit and adoption of poultry value chains enterprises among women managers [14].

Results further revealed that group membership increased the probability of adopting hybrid chicken by 8.7% among female managed chicken enterprises. Belonging to farmer chicken groups equip farmers with knowledge and skills on new agricultural practices and productive ventures such as hybrid chicken farming which boost their household incomes and ensure food security. Thus, need to empower women in the rural communities as they emerge to be the key stakeholders in driving the adoption of sustainable agricultural practices. The findings concur with those of [51] that found female chicken farmers group increased the probability of adopting improved chicken production technologies among smallholder farmers in Eastern Kenya. Furthermore, a study by [10] indicated that group membership offer platforms through which smallholder farmers can access information on agricultural technologies, access inputs and market their outputs.

Access to chicken feeds had a positive influence on the likelihood of adoption of hybrid chicken farming for the female decision maker. The ability of the female decision maker accessing the chicken feed increased their probability of adopting hybrid chicken by 15.1%. The possible reason is that female farmers often maximize the utilization of diverse feeds including locally available grains, kitchen leftovers, and other agricultural by-products to supplement the commercial feeds or formulate own feed mixtures. A study by [79] also found that inadequate and poor-quality feed resources is a constraining factor in livestock production among female- and male-headed households in Kenya.

An increase in the cost of the feeds by one unit increased the probability of adoption of hybrid chicken by 1.5% if the main decision maker of the enterprise was a male farmer but decreased their probability of adoption of hybrid chicken farming by 16.1% if the enterprise manager was a female farmer. Male farmers often have greater access to financial resources compared to their female counterparts. Males also generally have greater access to productive resources such as land ownership, access to credit and higher incomes [81–84]. With these resources at their disposal, male farmers may be better positioned to absorb the increased costs associated with adopting hybrid chicken breeds including purchasing higher-quality feed. The information obtained from surveyed farmers indicated that feed costs increased due to shocks such as COVID-19 pandemic and adverse effects of climate change in the region

Access to market had a positive influence on the likelihood of adoption of hybrid chicken farming if the main decision maker of the enterprise was a female. The ability of the female decision maker accessing market increased their probability of adoption of the hybrid chicken enterprise by 12.7%. The active involvement of female farmers in marketing of chicken suggests the key role they play in connecting chicken products to local and regional markets. Female farmers, as key decision makers in hybrid chicken farming, are not only producers but also key actors in the value chain, involved in activities such as selling live birds or eggs. These results agree with the findings of [45] and [85] who noted that ease of market access by female chicken enterprise managers increased the adoption of improved chicken production.

#### 3.2.2 Average treatment effects using propensity score matching.

Table 6 shows the propensity score matching estimates of the average treatment effect. The outcome variables considered in the study were the food consumption expenditure measured in Kenyan shillings spent per household and the food security status which was measured as a binary variable. The treatment variable in this case was the probability of the adoption of the hybrid chicken which was also measured as binary variable (1 = adopters and 0 = non-adopters). Following studies by [67] and [86], two matching algorithms which include the Nearest Neighbor Matching (NNM), and Radium Matching (RM) were used to calculate the Average Treatment Effects (ATT) based on the PSM model (Table 6). The results indicated a significant difference with ATT of 12 units in food consumption expenditure per household between adopters and non-adopters of the hybrid chicken enterprises managed by both male and female decision makers. This implied that male and female managers who adopted hybrid chicken saved KES 12 on household food consumption expenditure compared to non-adopters. The food security status for the households which reported to be at food surplus category was significantly different between adopters and non-adopters of the hybrid chicken farming.

**Table 6:** Propensity score matching estimates of the average treatment effect between adopters and non-adopters of hybrid chicken.

Mean outcome variables based matched observations				
Outcome variable	Matching algorithm	Adopters	non-adopters	ATT
Food Consumption expenditure	NNM	1180.54	1232.19	‒51.65**
	Radius	1192.34	1232.19	‒39.85**
Food security (1 = Food secure)	NNM	0.654	0.568	0.08
	Radius	0.721	0.568	0.15
Chronic food insecurity (yes)	NNM	0.025	0.028	‒0.01
	Radius	0.025	0.028	‒0.01
Transitory food insecurity(yes)	NNM	0.201	0.315	‒0.11
	Radius	0.201	0.307	‒ 0.10
Breakeven food security (yes)	NNM	0.531	0.512	0.02
	Radius	0.535	0.512	0.02
Food surplus(yes)	NNM	0.163	0.123	0.05**
	Radius	0.163	0.127	0.03**

**Note:**

***,

** and

*  denotes 1%, 5% and 10% levels of significance respectively, NNM=nearest neighbor matching; ATT is the average treatment effect, exchange rate at time of data collection 1USD = 120 Kenyan Shillings.

#### 3.2.3 ESR estimates of the average treatment effect.

The ESR model results on the effect of adoption of hybrid chicken on food consumption and household food security are presented in Table 7. The results indicated that adoption of hybrid chicken enterprise by male and female managers had a positive and significant influence on the household food security. This result corroborates with the findings of [30], who found that the adoption of hybrid chicken enhanced household food security and income among smallholder farmers in Kenya. On the food consumption expenditure, the results also showed that male and female managers who adopted hybrid chicken saved their consumption expenditure by KES 102 compared to them being non-adopters.

**Table 7:** Endogeneous switch regression estimates of the average treatment effect between adopters and non-adopters of hybrid chicken.

	HTTE	Decision stage		ATE
Outcome variable		To adopt	Not to adopt	
Food consumption expenditure	Adopters (ATT)	1192.13	1230.11	‒37.98***
	Non-adopters (ATU)	1210.18	1350.26	‒140.08***
Food security (1 = Food secure)	ATT	0.654	0.632	0.022**
	ATU	0.628	0.642	‒0.014***
Chronic food insecurity (yes)	ATT	0.029	0.041	‒0.012
	ATU	0.030	0.041	‒0.011**
Transitory food insecurity (yes)	ATT	0.295	0.315	‒0.020
	ATU	0.320	0.315	0.005
Breakeven food security (yes)	ATT	0.523	0.462	0.061
	ATU	0.538	0.451	0.087
Food surplus (1 = yes)	ATT	0.165	0.134	0.031***
	ATU	0.158	0.123	0.035***

**Note:**

***,

** and

*  denotes 1%, 5% and 10% levels of significance respectively, HTTE=Household type and treatment effect, ATE=Average treatment effect, ATT=Average treatment effect on treated; ATU=Average treatment effect on untreated, exchange rate at time of data collection 1USD = 120 Kenyan Shillings.

On the other hand, households with male and female managers as non-adopters had they decided to adopt the hybrid chicken enterprise, their household food consumption expenditures would have reduced by KES 102. These results are consistent with the findings of [67], who investigated the impact of adoption of improved maize varieties on household food security using endogenous switch regression model. The study found that if the adopting household had not adopted improved maize variety, their average household food consumption expenditure would have decreased and vice versa.

With respect to the food security aspect and the adopters and non-adopters of hybrid chicken farming with male and female managers, the results indicated that the probability of being food secure increased by 2.2% for the adopters of hybrid chicken between male and female managed enterprises. Similarly, the probability of being food secure for the non-adopters had they decided to adopt would be the same as the adopters of the hybrid chicken production if main decision maker were either male or female.

With regard to the chronically food insecure households, non-adopters of hybrid chicken production would have improved their food security index by 0.1% had they adopted hybrid chicken production. This result agrees with the findings of [87–89], who found that adopters who had embraced the modern agricultural technologies were more food secure compared to their non-adopters counterparts. Among the four categories of food security status, households who reported to be food secure indicated large probability differences. The results depict that for the households who were at food surplus category and adopted hybrid chicken production with male and female as the main decision makers increased their food security index by 3.1% and had the non-adopters in this category adopted hybrid chicken production they would further their indexes by 3.5%.

### 4.0. Conclusion and policy recommendations

This study employed quantitative methodologies to investigate the gender roles and constraints associated with hybrid chicken production and household food security in Lower Eastern, Kenya. PSM and ESR models were used to evaluate the determinants of adoption of hybrid chicken between male and female chicken enterprise decision-makers as well as average treatment effect for the effect of hybrid chicken production on household food consumption expenditure and food security. The results indicated that location of the farm, group membership, access to credit, access to market, access to feeds and feed costs significantly influenced the adoption of hybrid chicken. The study concludes that group membership, market outlets and access to credit play a key role in gendered decision making to adopt hybrid chicken production.

Furthermore, the results revealed that adoption of hybrid chicken among male and female managers positively impacted food nutrition security and reduced food consumption expenditures compared to their counterfactuals. To boost food security, this study recommends the National and County governments, in collaboration with development partners, to establish a supportive policy framework that promotes socially, environmentally and economically sustainable chicken production systems. In addition, there is need to enhance technical and extension services to facilitate cost effective chicken production, marketing, and disease management.

## Supplementary information

S1 Appendix Household questionnaire for chicken farmers in Machakos County. (DOCX)

S1 File Inclusivity-in-global-research-questionnaire. (DOCX)
