# Supplementary material for: Gender roles and constraints in enhancing hybrid chicken production for food security in lower Eastern Kenya
Source: PLoS One. 2025 Mar 3;20(3):e0318594. doi: 10.1371/journal.pone.0318594 (PMC11875333; doi:10.1371/journal.pone.0318594)
Supplement: S1 Appendix — (DOCX) [file pone.0318594.s001.docx]

**Questionnaire Proforma with Its List of Questions**

**HOUSEHOLD QUESTIONNAIRE FOR CHICKEN FARMERS**

**(PRIMARY RESPONDENT:** A person who makes most of the chicken production decision**)**

**Note to supervisors and enumerators: Both spouses to participate in the interview where possible**

| **Introductory statement and Consent:**  “Hello my name is……………................................................. a member of a team of researchers from the University of Embu, we are conducting a survey among chicken farmers to study production, marketing and livelihoods in your village. Your response to these questions would remain anonymous. Taking part in this study is voluntary. If you choose not to take part, you have the right not to participate and there will be no consequences. Thank you for your kind co-operation” |
| --- |

**MODULE A. HOUSEHOLD AND VILLAGE IDENTIFICATION**

| Sr.No. | Item | Response | Sr .No. | Item | Response |
| --- | --- | --- | --- | --- | --- |
| A1 | Name of the enumerator |  | A6 | Ward |  |
| A2 | Start time |  | A7 | Village |  |
| A3 | Household ID |  | A8 | GPS longitude |  |
| A4 | Survey Date (DD/MM/YY) |  | A9 | GPS Latitude |  |
| A5 | Sub county |  | A10 | GPS Altitude |  |

**MODULE B: HOUSEHOLD DEMOGRAPHICS AND HOUSING CONDITION.**

B1. Household size members_______________.

B2. Number of children below 18 years old in the HH_____________.

B3. Number of adults 18 years old and above in the HH­­­­­­­­­­­­­­­­­­ _____________.

B4. Indicate the following details for household head and spouse

|  |  | Name | Gender  0.Male  1.Female | Age (years) | Level of education  1.No formal education  2. primary  3.Secondary 4. Vocational  5.Tertiary | Marital status  1=Married  2=Divorced/ Separated  3=Widowed  4= Never married  5=Living together/De Facto | Main occupation  1-Farming  2-Formal Employment  3-Self-Employed  4.Casual Employment | Indicate the approximate household income in a month |
| --- | --- | --- | --- | --- | --- | --- | --- | --- |
|  | B4 | B.4.1 | B.4.2 | B.4.3 | B.4.4 | B.4.5 | B.4.6 | B.4.7 |
| B.4.1 | Head |  |  |  |  |  |  |  |

**B.5** Please observe the floor, roof and exterior walls of the main house and fill the following (**NOTE:** Ask only for ownership)

| Home ownership | Floor material | Wall material | Roofing material |
| --- | --- | --- | --- |
| B5.01 | B5.02 | B5.03 | B5.04 |
|  |  |  |  |
| Ownership codes/ B5.01  1=Owned 2=Borrowed  3=Rented 4=Other (specify) | Floor material codes/B5.02  1= Earth 2=Cement  3=Tiles 4=Other (specify) | Wall material codes/B5.03  1=Earth/mud 2=Cement/bricks  3=Wood/ iron sheets 4=Other, specify | Roof material codes/B5.04  1=Grass 2=Iron sheets  3=Tiles 4=Other (specify) |
|  |  |  |  |
|  |  |  |  |

**Module C: Resource Availability**

**C (1) Land Ownership and Use**

| Sr. No. | Item | Codes/Unit of measure | Response code |
| --- | --- | --- | --- |
| C1.1 | Total Land size | (Hectares) |  |
| C1.2 | Specify land size under agriculture | (Hectares) |  |
| C1.3 | Land tenure | 1=Owned with title deed 2= Owned without title deed  3= Rented/sharecropped 4= Owned by parent/relative  5= Borrowed |  |

**D. 3. Production**

**D.3.0.1** What is the type of chicken production enterprises owned? 1. Indigenous 2. Hybrid chicken 3. Both

**D. 3.1 Farm characteristics**

|  |  | Indigenous | Hybrid chicken |
| --- | --- | --- | --- |
| D.3.1.1 | Specify breed |  |  |
| D.3.1.2 | What is the number of chickens in this farm? |  |  |
| D.3.1.3 | Location of farm 1. Homestead 2. Another site |  |  |
| D.3.1.3 | Estimate the distance to the nearest input market in kilometers |  |  |
| D.3.1.4 | Do you use automated system on chicken farming? 1. Yes 2. No |  |  |
| D.3.1.5 | Is the farm able to access feed for the chicken 1. Yes 2. No |  |  |
| D.3.1.5 | Number of years in indigenous chicken farming |  |  |
| D.3.1.6 | Number of years in hybrid chicken farming |  |  |
| D.3.1.7 | Number of extension visits in a year specifically for chicken |  |  |
| D.3.1.8 | What is the cost of extension services provided in a month ? |  |  |
| D.3.1.9 | What is the cost of vaccination of chicken in a month ? |  |  |
| D.3.1.9 | What is the cost of feeds used in chicken per month? |  |  |
| D.3.1.10 | Did your household receive credit for chicken farming 1. Yes 2. No |  |  |
| D.3.1.11 | Are you a member or does any household member belong to chicken farming group ? 1. Yes 2. No |  |  |
| D.3.1.10 | If yes, how many chicken farming groups do you belong to? |  |  |

**SECTION E: Food Security**

**EI.1. Household Food Security (Food Consumption Score)**

**Consumption of Chicken and chicken products per month**

1. Number of chickens consumed by household per month……………………………….
2. Number of eggs consumed by household per month.........................................................

| Sr. No. | Food items | How many times in the last 7 days was the food consumed? |
| --- | --- | --- |
| E1.01 | Cereals & grains: Ugali, Githeri, mukimo, bread and mandazis |  |
| E1.02 | Roots & Tubers: potatoes, yams, cassava, white sweet potatoes. |  |
| E1.03 | Legumes& nuts: Beans, soy, pigeon pea, peanuts. |  |
| E1.04 | Vegetables: Carrots, red/yellow pepper (hoho), pumpkin, spinach, broccoli, amaranth, tomatoes, etc |  |
| E1.05 | Fruits: Mangoes, papaya, passion fruits, kiwi, apricot. |  |
| E1.06 | Meat/Fish/pork/beef/eggs/poultry: (in large quantities, not as condiments) |  |
| E1.07 | Milk &dairy products: Fresh milk, yoghurt, cheese |  |
| E1.08 | Oil/fat/butter: Vegetable oil and margarine |  |
| E1.09 | Sugar/sweets: Sugar, honey, jam, cakes, cookies, and sugary drinks. |  |
| E1.10 | Condiments/spice: Tea, coffee, cocoa, salt, garlic, yeast /backing powder, tomato sauce, meat /fish condiments, others |  |
| E1.11 | What was the main source of food for the past 7 days?  1=Own production 5=Gifts  2=Purchase (cash) 6= Exchange for labour  3 Purchase (credit) 7= Others, specify  4=Food assistance |  |

**E.2 Food Insecurity Experience Scale (FIES)**

| Sr. No. | Situation | During the last 12 months was there a time when (Situation)? | | How often did this (Situation) happen in the past 12 months?  1=None 2=Rarely (1 -2 times) 3=Sometimes (3 -10 times) 4=Often (>10 times) |
| --- | --- | --- | --- | --- |
| E2.01 | You were worried that any household members would not have enough food to eat because of lack of money or other resources? | |  |  |
| E2.02 | You or any household members were not able to eat the healthy and nutritious foods because of lack of money or other resources? | |  |  |
| E2.03 | You or any household members ate a few kinds of foods because of lack money or resources? | |  |  |
| E2.04 | You had to skip a meal because there was no enough money or resources to get food? | |  |  |
| E2.05 | You or any household members ate less than you thought you should because of lack of money or other resources? | |  |  |
| E2.06 | There was no food at all in your house because of lack of money? | |  |  |
| E2.07 | You or any household member did not eat because there was not enough money or other resources for food? | |  |  |
| E2.08 | You or any household members went without eating for a whole day because of lack of money or resources? | |  |  |
|  | General Food security status | |  | 1. Chronic Insecure 2. Transitory Insecure 3. Breakeven food secure 4. Food Surplus |

**E.3 Household Dietary Diversity Score (HDDS)**

Now I would like to ask you about the types of foods that you or anyone else in your household ate specifically in the last 24 hour. Please, tell me by accurately recalling

**Ask the two columns separately. For 24 hr recall try to ask what was eaten at each meal time and list them on the left side of the table and sort them based on the food groups**

**Column 3 &4:** *Read the list of foods. Place a “1” in the box if anyone in the household ate the food in question; or place a “0” in the box if no one in the household ate the food*

| Sr.No. | FOOD | Description | During the last day and night | During the last day and night |
| --- | --- | --- | --- | --- |
|  |  |  | Child | Mother |
| E3.01 | Cereals | Corn/maize, rice, barley, oats, wheat, sorghum, finger millet or any other grains or foods made from these (e.g. bread, biscuits, noodles, porridge, or other grain products) |  |  |
| E3.02 | Vitamin a rich vegetables and tubers | Pumpkin, carrots, squash, or yellow/orange flesh sweet potatoes or *other locally available vitamin A rich vegetables (e.g. red/yellow sweet pepper)* |  |  |
| E3.03 | White tubers and  roots | White potatoes, white yams, white cassava, or other foods made from roots |  |  |
| E3.04 | Dark green leafy  vegetables | Dark green/leafy vegetables, including wild ones + *locally available vitamin A rich leaves such as amaranth,Cassava leaves, Kale, Spinach, brocoli etc.* |  |  |
| E3.05 | Other vegetables | Other vegetables (e.g. tomato, onion, eggplant) , including wild vegetables |  |  |
| E3.06 | Vitamin a rich fruit | Ripe mangoes, cantaloupe, apricots (fresh or dried), Ripe papaya, dried peaches, passion fruits, kiwi, melon, guavas |  |  |
| E3.07 | Other fruits | Other fruits, including wild fruits (e.g pears, banana, apples, lemon, plums, grapes and tangerine |  |  |
| E3.08 | Organ meat /(iron rich) | Liver, Kidney, Heart or other organ meats or blood-based foods (e.g………………………….) |  |  |
| E3.09 | Flesh meats | Beef, pork, lamb, goat, wild game, chicken, or other birds |  |  |
| E3.10 | Eggs | Chicken, duck, guinea hen or any other egg |  |  |
| E3.11 | Fish | Fresh or dried fish or shellfish |  |  |
| E3.12 | Legumes, nuts and  seeds | Beans, peas, lentils, nuts, soy, lentils, seeds or foods made from these |  |  |
| E3.13 | Milk and milk  products | Milk, cheese, yogurt or other milk products |  |  |
| E3.14 | Oils and fats | Oil, fats or butter added to food or used for cooking, oil seeds and foods made from oil seeds e.g. sunflower |  |  |
| E3.15 | Red palm products | Red palm oil, palm nut or palm nut pulp sauce |  |  |
| E3.16 | Sweets | Sugar, honey, sweetened soda or sugary foods such as chocolates, candies, cookies and cakes |  |  |
| E3.17 | Spices, condiments,  beverages | Spices (black pepper, salt), condiments (soy sauce, hot sauce), coffee, tea, |  |  |
| E3.18 | Meal outside home | Did you or anyone in your household eat anything (meal or snack) outside of the home yesterday? |  |  |

**G4: Food and Non-Food Expenditure**

How much did you spend on the following per month **(KES)**

| Sr. No. | Monthly expenditure | Amount (KES) |
| --- | --- | --- |
| G4.01 | Food expenses (salt, sugar, beverages etc) |  |
| G4.02 | Non-food expenses (clothes, airtime, transport, etc) |  |
| G4.03 | Hospital fee and buying medicine |  |
| G4.04 | School fees |  |

**Module F: Gender Roles in Chicken Production**

**Gender participation in chicken production activities (Please Tick where appropriate)**

| Activity | Male | Female |
| --- | --- | --- |
| Construction of chicken houses 0 No 1 Yes |  |  |
| Feeding of the poultry 0 No 1 Yes |  |  |
| Vaccination of the chicken 0 No 1 Yes |  |  |
| Buying of equipment’s 0 No 1 Yes |  |  |
| Supply of breeding stock 0 No 1 Yes |  |  |
| Trading of chicken beyond the farm-gate 0 No 1 Yes |  |  |
| Marketing of chicken locally 0 No 1 Yes |  |  |
| Security 0 No 1 Yes |  |  |
